# Inhibiting MicroRNA-141-3p Improves Musculoskeletal Health in Aged Mice

**DOI:** 10.14336/AD.2023.0310-1

**Published:** 2023-12-01

**Authors:** Sagar Vyavahare, Sandeep Kumar, Kathryn Smith, Bharati Mendhe, Roger Zhong, Marion A. Cooley, Babak Baban, Carlos M. Isales, Mark Hamrick, William D Hill, Sadanand Fulzele

**Affiliations:** ^1^Department of Cell biology and Anatomy, Augusta University, Augusta, GA, USA.; ^2^Department of Physiology & Cell Biology, University of Arkansas for Medical Sciences, Arkansas, USA.; ^3^Department of Neuroscience and Regenerative Medicine, Augusta, GA, USA.; ^4^Department of Oral Biology and Diagnostic Sciences, Augusta University, Augusta, GA, USA.; ^5^Department of Medicine, Augusta University, Augusta, GA, USA.; ^6^Center for Healthy Aging, Augusta University, Augusta, GA, USA.; ^7^Department of Neuroscience and Regenerative Medicine, Augusta, GA, USA.; ^8^Department of Pathology and Laboratory Medicine, Medical University of South Carolina, SC 29403, USA.

**Keywords:** miR-141-3p, anti-mir, aging, senescence, inflammation, AUF-1

## Abstract

Emerging evidence shows that the microRNA-141-3p is involved in various age-related pathologies. Previously, our group and others reported elevated levels of miR-141-3p in several tissues and organs with age. Here, we inhibited the expression of miR-141-3p using antagomir (Anti-miR-141-3p) in aged mice and explored its role in healthy aging. We analyzed serum (cytokine profiling), spleen (immune profiling), and overall musculoskeletal phenotype. We found decreased levels of pro-inflammatory cytokines (such as TNF-α, IL-1β, IFN-γ) in serum with Anti-miR-141-3p treatment. The flow-cytometry analysis on splenocytes revealed decreased M1 (pro-inflammatory) and increased M2 (anti-inflammatory) populations. We also found improved bone microstructure and muscle fiber size with Anti-miR-141-3p treatment. Molecular analysis revealed that miR-141-3p regulates the expression of AU-rich RNA-binding factor 1 (AUF1) and promotes senescence (p21, p16) and pro-inflammatory (TNF-α, IL-1β, IFN-γ) environment whereas inhibiting miR-141-3p prevents these effects. Furthermore, we demonstrated that the expression of FOXO-1 transcription factor was reduced with Anti-miR-141-3p and elevated with silencing of AUF1 (siRNA-AUF1), suggesting crosstalk between miR-141-3p and FOXO-1. Overall, our proof-of-concept study demonstrates that inhibiting miR-141-3p could be a potential strategy to improve immune, bone, and muscle health with age.

## INTRODUCTION

Aging is a complex physiological process characterized by gradual and progressive degradation, loss of function, and reduction in the repair capacity of the tissues. Eventually, this leads to multiple age-related complications such as cardiovascular, Parkinson's, dementia, osteoporosis, and sarcopenia [1]. Chronic oxidative and inflammatory stress are important factors in age-related dysfunction [2]. Several studies demonstrated elevated serum pro-inflammatory cytokines and oxidative stress with age [3-5]. Oxidative stress and inflammatory factors regulate senescence-related factors (e.g., p21, p16), which induce cells to undergo premature senescence. The age-related degenerative process is complicated due to the involvement of multiple factors (e.g., histone modification, methylation, miRNAs).

Recent studies suggested that microRNAs (miRNAs) play a crucial role in age-related degenerative diseases. For example, the microRNAs miR-21, miR-22, miR-34a, and miR-17 are altered in age-related pathologies [6-9]. Several studies reveal the involvement of microRNAs (miR-181a, miR-434-3p, miR-431, miR-29, and miR-126) in the skeletal muscle aging process [10-12]. The group of miRNAs such as microRNA-19b-3p (Liu et al., 2022) [13], miRNA-365a-3p [14], miRNA-577 [15], and miRNA-338-3p [16] is involved in the age-related bone loss.

MicroRNAs are small non-coding RNAs that bind to 3′-untranslated regions (3′-UTRs) of mRNA to cause mRNA degradation or block translation [17]. MicroRNAs are known to be involved in influencing several aging-related signaling pathways, such as insulin-like growth factor signaling, a target of rapamycin (TOR), reactive oxygen species (ROS) signaling, DNA damage response, and mitochondrial dysfunction [7,18,19] Previously, our and other studies demonstrated elevated levels of miR-141-3p with age and its involvement in bone marrow stromal cell (BMSCs) differentiation [20-23]. We reported that microRNA 141-3p is elevated in aged human and murine bone marrow microenvironments. We also reported that miR-141-3p regulates the expression of SDF1 and vitamin C transporter (sodium-dependent vitamin C transporter 2 (SVCT2) in BMSCs. Furthermore, we demonstrated that inhibition of miR-141-3p increases the osteogenic differentiation of BMSCs. The underlying mechanism of miR-141-3p remains elusive as to what extent miR-143-3p is involved in the aging process. Based on ours and other studies, we hypothesize that inhibition of miR-141-3p in aged mice might delay age-related complications and improve systemic health.

Given the current scenario and the trend in the mRNA (mRNA Vaccine) and microRNA research, the therapeutic potential of microRNAs is being harnessed to treat age-associated degenerative diseases [24, 25]. MicroRNA mimics can be synthetic and used to restore the level of beneficial microRNAs, which reduce along with age. Similarly, synthetic antagomirs can be used for anti-aging potential, which reduces the level of elevated microRNAs with age. In this study, we treated aged mice of both sexes with Anti-miR-141-3p for three months and analyzed the overall health. Furthermore, we identified miR-141-3p dependent signaling pathways involved in muscle biology.

## MATERIAL AND METHOD

### Animal Handling

All the animal protocols were approved by Institutional Animal Care and Use Committee at Augusta University. Male (n=20) and female (n=20) mice (C57BL6) were obtained at 14 months and housed in a 12-h light/dark cycle and had free access to food and water throughout the study. After acclimatizing an animal facility environment (~one Month), the Anti-miR-141-3p (n=20, male 10, female 10, 40nM/kg/body weight, Cat#: MIN0000519) and Scrambled miR (Vehicle/Control n=20, male 10, female 10, Cat#: 339122YI04100685-AFA, Qiagen, USA); were administered subcutaneously twice a week for 12 weeks. Desired concentration (40nM) of Anti-miR-141-3p was prepared by using DOTAP liposomal transfection reagent (Cat#: 11202375001, Sigma, USA) followed by diluting with Phosphate Buffer Saline (PBS). Similarly, Scrambled miR was prepared and used to inject into the control cohort. The mice were sacrificed at around eighteen months to collect blood, serum, femur, tibia, and muscles. The tibia and femur were excised carefully, and all the soft tissue was removed from around the bone. Muscle: tibialis antaralis was collected for RNA and protein, while quadriceps femoris was collected for immunohistochemical analysis. Furthermore, the femurs were fixed in 4% paraformaldehyde for micro-CT.

### Serum cytokine Analysis

The LEGEND plex mouse Inflammation Panel with V-bottom Plate (BioLegend Cat# 740446) was used to quantify cytokines in mouse serum according to our published method [26] and manufacturer's instructions. The LEGENDplex mouse Inflammation Panel with V-bottom Plate (BioLegend Cat. No. 740446) was used to perform simultaneous cytokine measurements in mouse serum. The samples were thawed and centrifuged to eliminate debris/particles. Samples were diluted 2-fold with assay buffer for measurement accuracy, and standards were blended with Matrix C (BioLegend). Standards and samples were plated with capture beads and incubated at room temperature for 2 hours on a plate shaker (800 rpm). Detection antibodies were added to each well after washing the plate with a wash buffer. The plate was incubated on a shaker for one hour at room temperature. Finally, streptavidin phycoerythrin (SA-PE) was added without being washed and incubated for 30 minutes. The CytoFLEX flow cytometer (Beckman Coulter Life Sciences, Indianapolis, IN, USA) was used to collect samples. The R package DrLumi [27] installed on R 3.5.2, was used to create standard curves and protein concentration. The detection limit was obtained by taking the average of the background samples + 2.5 x SD. The Immune Monitoring Shared Resource (Augusta University) was used to perform the assay and data computations.

### Micro-Computed Tomography analysis (μCT)

To measure bone mineral density and 3D morphometric analysis, femurs were fixed in 10% formalin and scanned in μCT system (Skyscan 1272; Bruker MicroCT, Kontich, Belgium) as previously described [28]. In brief, scanning was performed at an image pixel size of 9.5 μm2 at a resolution of 1224x820 pixels. Projection images were acquired using an X-ray source voltage of 70 kV at 142 μA through a 0.5 mm aluminum filter. Each sample was rotated 180° in 0.5° steps with four averaged frames per step. Projections were reconstructed using NRecon software (ver. 1.7.4.6). Then the reconstructed data sets were loaded into the Bruker CT-Analyser program (ver. 1.20.8) for 3D morphometric parameters and bone mineral density measurements. After calibrating with hydroxyapatite phantoms of known density, a zone of interest matching the femoral head and neck was selected, and bone mineral density was determined in each region of interest.

### Histological staining

Immediately after euthanizing the mice, quadriceps muscles were dissected and placed in 10% buffered formalin. The samples were embedded in paraffin, cut in cross-sections, and stained for hematoxylin and eosin to measure fiber size. Muscle fiber size was calculated by creating a grid using ImageJ, and one muscle fiber was measured in each voxel. Sections were selected randomly from each mouse (n=20/group), and 25 muscle fibers per section were counted per mouse to calculate an average fiber size per muscle. Using a brightfield Leica DMCS microscope equipped with a micropublisher six camera (QI Imaging), photos from standardized sections of each cross-section were taken at 20x magnification and used to compute fiber size. To avoid any bias, all measurements were performed by an investigator blinded to group assignment. To carry out laminin staining, the sections were deparaffinized, followed by incubation with Digest All (Thermo Fisher Scientific Cat No. 003009) in the oven for 10 min using a humidified chamber. Then, the sections were blocked with serum-free protein block (Dako Cat No. X0909) for 20 min. Anti-laminin (Sigma L9393) primary antibody (50 μl/section) at 1:25 dilution was added to the sections and incubated for 2 hours at room temperature, followed by incubation with secondary antibody-Alexa Flour 546 goat anti-rabbit (Life Technologies A11010) at 1:250 dilution for 1 hour in the dark. Vectashield mounting medium with DAPI (Vector H-1500) 50 μl/section was added to the sections, and imaging was performed using Leica Stellaris confocal microscope. Five photographs were taken using a 20× lens for each section.

### In Vitro Studies/Cell culture studies

C2C12 cells were procured from ATCC (ATCC® CRL-1772™) for *in-vitro* studies. The cells were cultured in Dulbecco’s Modified Eagle’s Medium (DMEM) (Gibco, USA) containing 10% fetal bovine serum (FBS) (Gibco, USA) and 1% penicillin-streptomycin (Gibco, USA) at 37° C in a 5% CO2 cell incubator (Thermo, USA) until 70%-80% confluence. The microRNA-141-3p mimic (Cat#: No: MSY0000153, Qiagen, USA) and inhibitor of miR-141-3p (Cat#: No: MIN0000519, Qiagen, USA) were obtained from QIAGEN. The transfection of miRNA mimics and inhibitors was performed using a lipofectamine transfection reagent (Lipofectamine 2000, Invitrogen 11668019) according to the manufacturer's procedure. In brief, C2C12 cells were seeded at 500K cells in 100mm plates followed by transfection of mimic and inhibitor (20 μM mimic or 40 nM inhibitor as final concentration) at 70-80% confluence in 2% FBS, DMEM culture media. After 48 hours, cells were used for western blot, RNA isolation, and senescence assay.

### Isolation of RNA, synthesis of cDNA, and real-time PCR

Total RNA was isolated from the mice muscle. The muscle was washed with 1X PBS, chopped, minced, and homogenized in Trizol. MicroRNA was isolated from the cell lysates using the miRNA easy isolation kit and reverse transcribed into cDNA using miScript II RT kit (Qiagen, Cat. No 218160). Fifty pictograms of cDNA were amplified in each qRT-PCR using SYBR Green I (Biorad, Hercules, California, USA) and miR-141-3p-specific primers (Qiagen, USA). The RNU6 (RNA, U6 small nuclear 2) was used as the internal control for normalization.

### Western blot analysis

At the end of experiments, proteins were extracted from cells and muscle using RIPA-cell lysis buffer containing protease inhibitor (Invitrogen 89900), subjected to SDS-PAGE, and transferred to nitrocellulose membrane. To prevent non-specific binding, blocking was performed with 5% dried fat milk in 1× PBST (Phosphate Buffered Saline with Tween 20). Then the membranes were incubated with antibodies against AU-rich element RNA-binding protein 1 (AUF-1) [Cat#: 12382S], Forkhead Box O1 (FOXO-1) [Cat#: 2880S],, Tumor necrotic factor (TNF-α)(Cat#: 3707, Cell Signalling Technology, Danvers, MA ), p-16 (Cat#: 80772S), p-21(Cat#:2947S), Interleukin 1-β (IL-1β)(Cat#: 12703S) (Cell Signalling Technology, Danvers, MA), GAPDH (Cat#: 97166S, Cell Signalling Technology, Danvers, MA) and beta-actin (Cat#: SC4778, Santa Cruz, CA) overnight at 4ºC, followed by incubation with HRP-conjugated goat anti-rabbit IgG antibody/anti-mouse IgG antibody. Proteins were visualized with an ECL Western blot detection system (ChemiDoc, Biorad). GAPDH and beta-actin were used as the loading control.

**Figure 1.**
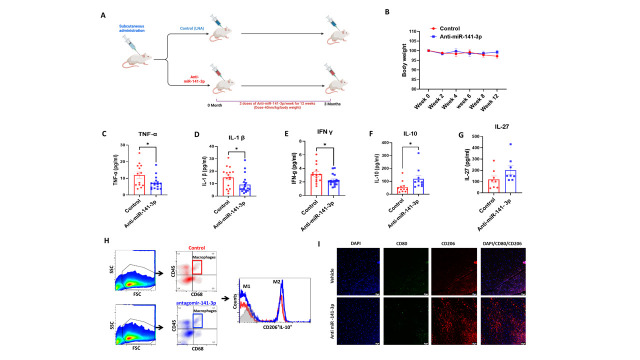
Inhibiting miR-141-3p reduces systemic inflammation in aged mice. (A) Experimental design for the study of the anti-miR-141-3p regime. In the current study mice male and female (n=18-20/ group) of 15-month age were injected with Anti-miR-141-3p (40 nM) twice a week subcutaneously for three months. (B) No significant change was observed in body weight. Serum cytokine analysis demonstrated significantly lower levels of pro-inflammatory cytokines (C) TNF-α (p<0.028), (D) IL-1β (p<0.030), (E) IFN-γ (p<0.022), (F) IL1-10 was augmented (p<0.034) with the same trend observed in g) IL-27. Spleen flow cytometry data analysis revealed an increased (H) M2 macrophage population (CD206) and an increase in anti-inflammatory cytokine IL-10, suggesting anti-miR-141-3p reduces inflammation with the advancement of age. (I) Double immunostaining on muscle sections showed increased staining for CD206 (M2) macrophage populations than M1 (CD80) in the treatment group compared to the vehicle-treated. Data are expressed as mean ± SEM. Data were analyzed by student’s t test for two groups or ANOVA for more than two groups (n=18-20/group *p<0.05, **p<0.01, #p<0.001).

### Cellular senescence assay

C2C12 cells were seeded in 24 well plates and treated with miRNA mimic and Anti-miR-141-3p, as mentioned above to assess SA-beta-galactosidase (SA-β gal). After 48 hours of treatment, senescence quantification was performed according to the manufacturer's instructions (ENZKIT1300010). Cell lysate, along with an equal volume of freshly prepared reaction buffer (2X assay buffer included 10mM of beta-mercaptoethanol and 1 X beta-gal substrate) was incubated for 2 hours at room temperature. The reading was performed using a plate reader (Epoch, Biotek) at 360nm (Excitation) and 465nm (Emission).

**Figure 2.**
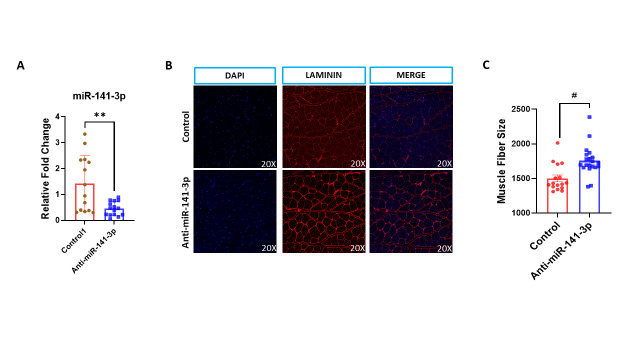
Inhibition of miR-141-3p improves muscle fiber size. Quadriceps muscle was collected from the right leg and fixed in formalin. H&E staining was performed to determine muscle fibre size. 25 different areas were measured on each slide. Anti-miR-141-3p treated mice showed a decrease in (A) miR-141-3p expression and an increase in (B, C) muscle fiber size (p<0.001). Laminin staining demonstrated that Anti-miR-141-3p augmented muscle fiber Size. Data are expressed as mean ± SEM. Data were analyzed by student’s t test for two groups or ANOVA for more than two groups (n=18-20/group *p<0.05, **p<0.01, #p<0.001).

### Myoblast differentiation Assay

Transfected C2C12 cells were seeded (1x10^5^) in 4 well chamber slides (Falcon Ref 223522) and cultured in DMEM media with 10% FBS. Once the cells reached 80% confluence, the media was changed to DMEM with 2% horse serum to induce differentiation. Cells were treated every other day in differentiation media (2% horse serum) for 96 hours with miR-141-3p mimic and anti-miR-141-3p. Five photographs were taken using a 20× lens for each well using a Leica DMi1 Flexacam-C1 microscope.

### Flow cytometry on Splenocytes

Spleens were harvested and placed in RPMI + 10% FBS media. Single-cell suspensions were prepared using Collagenase IV (100 CD units/ml) (Sigma, St Louis, MO) followed by mincing, and cells were washed twice with PBS. Next, spleen cells were incubated with antibodies for surface markers (CD68, CD45, CD206) for 20 minutes on ice in the dark (all antibodies from Pharmingen-BD-Biosciences, San Jose, CA). Cells were washed with PBS, fixed, and permeabilized with Fix/Perm Buffer (eBiosciences, San Diego, CA) and kept on ice for 15 minutes. Followed by intracellular staining with IL-10 antibody for 20 minutes in the dark (BD Biosciences). Then, cells were washed, and flow cytometry (FACS Calibur, Becton Dickinson) was performed. Dead and debris cells were excluded by gating and side scatterplots. For analysis, 100,000 total events were collected, and data were represented as a percentage of the number of gated event cells expressing a specific marker.

### Immunofluorescent staining of macrophage

The tissue sections were deparaffinized, followed by antigen retrieval. Sections were washed in 0.1% Triton X twice for 10 minutes and blocked using 2% bovine serum albumin (BSA) for one hour at room temperature. After blocking, sections were incubated with primary antibodies (1:100) recognizing CD206 (Thermo scientific Cat#: PA5-101657) and CD80 (Polyclonal Goat IgG, Cat#: AF740-SP R&D Systems) overnight at 4°C. The sections were rinsed three times with PBS and then incubated for 1 hour at room temperature with secondary antibody (Donkey Anti-Rabbit Alexa Fluor 647 Cat#: A32795, and Donkey Anti-Goat Alexa Fluor 488 Cat#: A-11055, ThermoFisher Scientific). Then the sections were mounted using a mounting medium with DAPI (Vector H-1500) and imaged with a Leica Stellaris confocal microscope. Five photographs were taken using a 40× lens for each section. Cells stained red (CD206) were counted as M2 macrophages, whereas cells stained with green (CD80) were counted as M1 macrophages. Cells that were stained yellow (red + green; CD206 + CD80) were counted as total macrophages.

### Statistical analysis

The results were shown as mean ± standard deviation. A p-value of <0.05 was considered significant. GraphPad Prism 8 (La Jolla, CA) was utilized to perform ANOVA with Bonferroni pair-wise comparison or unpaired Student’s t-tests as appropriate. The non-parametric Mann-Whitney U test was used to compare non-parametric datasets (non-normal distribution or n<6)

**Figure 3.**
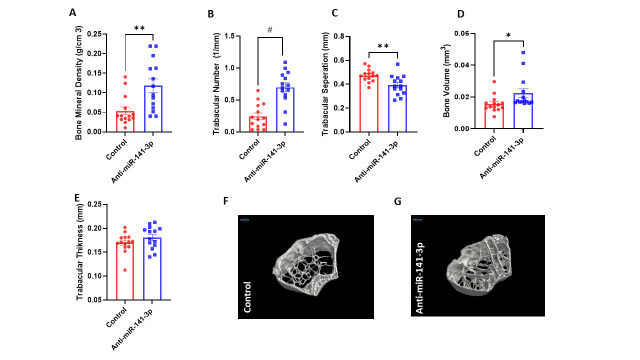
Inhibition of miR-141-3p improves bone microstructure: Microcomputed tomography (μCT) on mouse femurs showed significant improved (A) bone mineral density (p<0.003), (B) trabecular number (p<0.001), (D) bone volume (p<0.037) and decreased (C) trabecular separation (p<0.004) with anti-miR-141-3p treatment as compared to vehicle control. Data are expressed as mean ± SEM. Data were analyzed by student’s t test for two groups or ANOVA for more than two groups (n=18-20/group *p<0.05, **p<0.01, #p<0.001).

## RESULTS

### Inhibiting miR-141-3p reduces inflammation in aged mice

**Figure 4.**
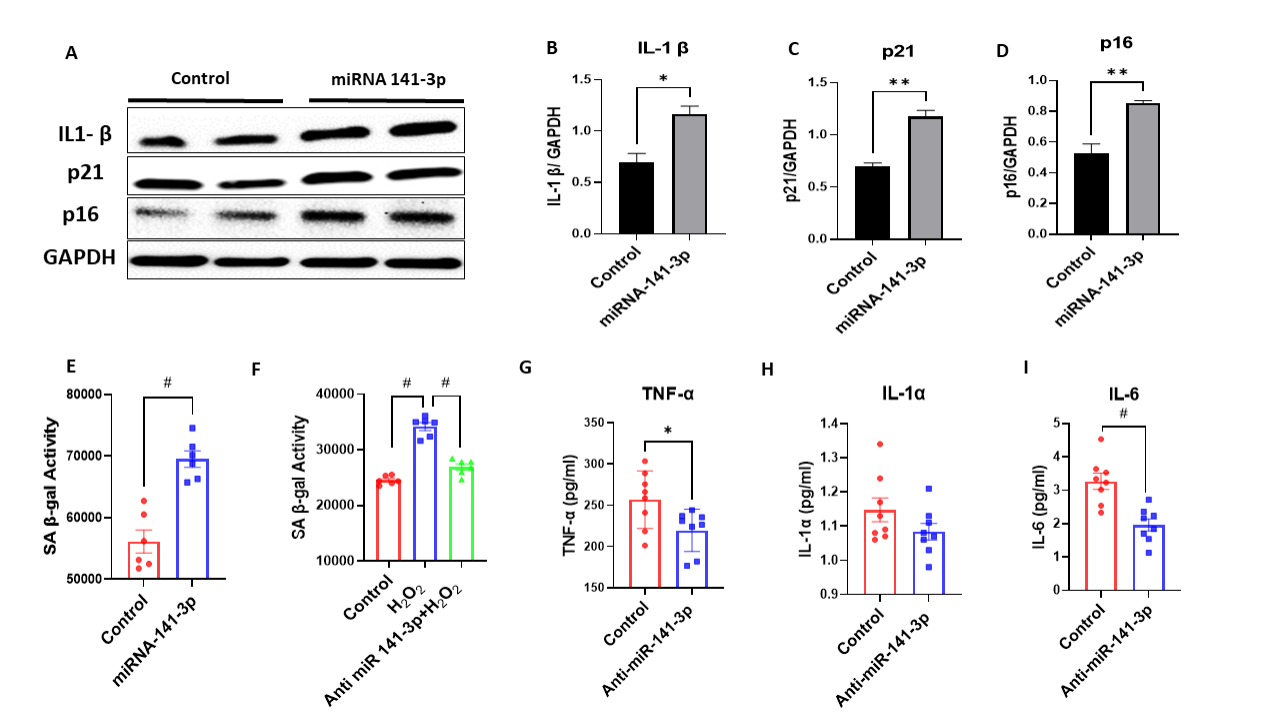
MicroRNA-141-3p regulates cellular senescence (SASP-related proteins) in C2C12 cells. Western blot analysis revealed that C2C12 cells treated with miR-141-3p mimic showed enhanced expression of SASP-related proteins (A) IL-1β, p21 and p16. The panel (B)) IL-1β (p<0.017), (C) p21 (p<0.002), and (D) p16 (p<0.006) showing quantification of western blot. The panel (E) showing miRNA-141-3p upregulated the SA-β gal activity and (F) anti-miR-141-3p (p<0.002) reduces the oxidative stress induced SA-β gal activity (p<0.001) in C2C12 cells. Anti-miR-141-3p treatment also reduces the proinflammatory cytokines (G) TNF-α (p<0.0292) (H), IL-1α (p<0.155) and i) IL6 (p<0.007) in conditioned culture media of C2C12 cells (n=8/group *p<0.05, **p<0.01, #p<0.001). Data are expressed as mean ± SEM. The non-parametric Mann-Whitney U test was used to compare non-parametric datasets (non-normal distribution or n<6). Data were analyzed by student’s t test for two groups or ANOVA for more than two groups (n=4-6/group *p<0.05, **p<0.01, #p<0.001).

It is well known that with advanced age, systemic inflammation is elevated. We analyzed serum cytokine levels and spleen immune profiling. We found that the levels of pro-inflammatory cytokines such as TNF-α (p<0.028) (Fig. 1C), IL-1β (p<0.030) (Fig. 1D), and IFN-γ (p<0.022) (Fig. 1E) were significantly decreased in the mice treated with anti-miR-141-3p while IL-10 was augmented (p<0.034) (Fig. 1F) with the same trend observed in IL-27 (Fig. 1G). Flow cytometry data from the spleen also showed similar outcomes. Anti-miR-141-3p treated splenocytes showed a significant (p<0.05) decrease in TNF-α expressing macrophages (M1-polarized macrophages) and an increase in IL-10-expressing (p<0.04) macrophages (M2-polarized macrophages). A significant (p = 0.005) polarization from predominately M1 to M2 macrophage was also observed in splenic tissue of Anti-miR-141-3p treated animals (Fig. 1H). The *in-vitro* studies on C2C12 cells showed decreased pro-inflammatory cytokines with Anti-miR-141-3p treatment. We treated C2C12 cells with anti-miR-141-3p and used conditioned culture media for cytokine analysis. We found a significant decrease in the levels of pro-inflammatory cytokines such as TNF-α (p<0.0290) (Fig. 4G) and IL-6 (p<0.007) (Fig. 4I). We also found a decreasing trend of IL-1α levels in Anti-miR-141-3p treatment (Fig. 4H). Moreover, we also validated cytokines levels in the muscle lysate of Anti-miR-141-3p treated animals. We found that IL-1β levels were decreased (p<0.096) in Anti-miR-141-3p treated animals compared to the vehicle-treated group (Fig. 6D). Moreover, we also performed immunostaining on muscle sections to identify the M1 and M2 macrophage markers. The muscle sections were double immunostaining using antibodies that recognized the M1 marker (CD80) and M2 marker (CD 206). We observed an increased number of CD206 (M2) macrophage populations than M1 (CD80) in the treatment group compared to the vehicle-treated (Fig. 1I).

### Inhibition of miR-141-3p improves musculoskeletal health

Previously, several studies demonstrated elevated levels of miR-141-3p in musculoskeletal complications [29, 30]. We hypothesized that inhibiting miR-141-3p with age might improve musculoskeletal health. To test this hypothesis, anti-miR-141-3p was administered subcutaneously in both male and female mice twice a week for three months to test the effect of anti-miR-141-3p on musculoskeletal health. Muscle fiber size was significantly higher (p<0.001) in anti-miR-141-3p treated mice as compared to the control group (Fig. 2C). To confirm our findings, we performed laminin staining. Laminin staining showed similar results, indicating that anti-miR-141-3p treated mice showed increased muscle fiber size (Fig. 2B). Furthermore, the miR-141-3p analysis on muscle lysate showed significant downregulation (p<0.01) in anti-miR-141-3p treated animals (Fig. 2A). The bone microarchitecture data showed a significant increase in bone mineral density (p<0.003) (Fig. 3A), trabecular number (p<0.001) (Fig. 3B) and bone volume (p<0.03) (Fig. 3D) and decrease in trabecular separation (p<0.004) (Fig. 3C) in anti-miR-141-3p treated mice. Our data suggested that inhibiting miR-141-3p improves musculoskeletal health by improving bone microstructure (Fig. 3) and muscle fiber size (Fig. 2).

#### Anti-miR-141-3p induces myogenic differentiation and reduces premature senescence

C2C12 cells were transfected with mir-141-3p mimic or anti-miR-141-3p to evaluate their role in myogenic differentiation. Our results showed that miR-141-3p mimic inhibited myogenic differentiation, and anti-miR-141-3p showed improved myogenesis (Supplementary Fig. 1).

Senescence-associated secretory phenotype (SASP) inflammatory cytokines are elevated with age [31]. Our data showed decrease IL-1β levels in serum and muscle protein lysate in the anti-miR-141-3p treated group. We hypothesize that miR-141-3p might play an important role in inducing premature senescence, and the anti-miR-141-3p can prevent or reduce this process. We treated C2C12 cells with miR-141-3p mimic and anti-miR-141-3p and performed cellular senescence activity assay and western blot on p16 and p21. We found increased SA-β-gal activity in the miR-141-3p treated group compared to the control (Fig. 4E), and anti-miR-141-3p treatment reduces the oxidative stress (H2O2) induced β-gal activity (Fig. 4F). Western blot analysis also showed a significant increase in senescence markers such as p16 (p<0.006) (Fig. 4D) and p21 (p<0.002) (Fig. 4C) with miR-141-3p mimic and decreased with anti-miR-141-3p treatment. Furthermore, we performed a western blot on senescence markers (p16 and p21) on muscle cell lysate. We found significantly reduced expression of p16 (p<0.001) (Fig. 6F) and p21 (Fig. 6E) in anti-miR-141-3p treated group compared to the vehicle-treated.

**Figure 5.**
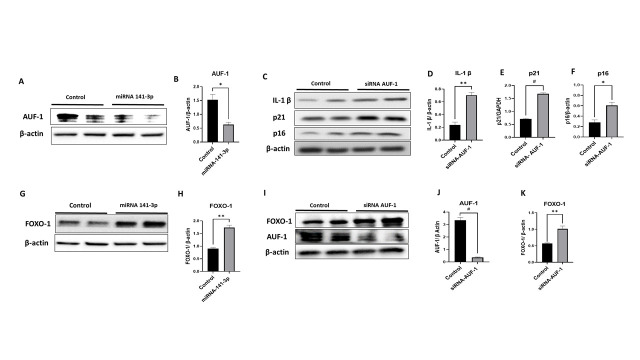
MicroRNA-141-3p and RNA binding protein AUF1 regulate the SASP-related proteins and FOXO1 expression in C2C12 cells. The representative (A) Western blot and (B) quantification analysis revealed that miRNA-141-3p reduced AUF1 expression (p<0.013) in C2C12 cells. The inhibition of AUF1 using siRNA elevated the expression of (C) SASP-related proteins, (D) IL-1β, (E) p21, and (F) p16 in C2C12 cells (*n=5-6/group* *p<0.05, **p<0.01, #p<0.001). The representative (G) western blot and (H) quantification of (western blot) showing mir-141-3p augmented the expression of FOXO-1 (p<0.001). The suppression of AUF-1 using siRNA iI &J) decrease AUF1 (p<0.001) and increases (I & K) FOXO-1 (p<0.01) expression (*n=4-6/group* **p<0.01, #p<0.001). Data are expressed as mean ± SEM. The non-parametric Mann-Whitney U test was used to compare non-parametric datasets (non-normal distribution or n<6). Data were analyzed by student’s t test for two groups or ANOVA for more than two groups (n=4-6/group *p<0.05, **p<0.01, #p<0.001).

### miRNA-141-3p regulates AUF1-FOXO-1 signaling

**Figure 6.**
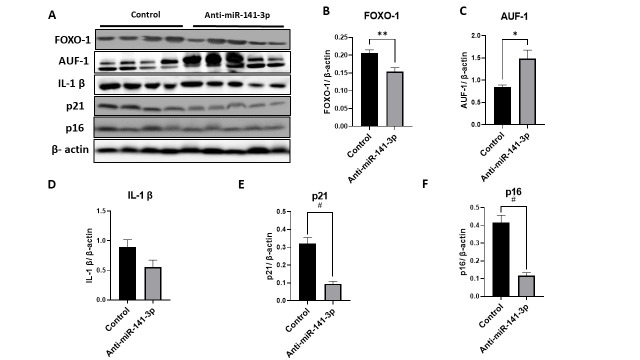
*In-vivo* inhibition of miR-141-3p improves muscle health by suppressing SASP and FOXO-1 protein expression. Representative image of western blot from muscle lysates of anti-miR-141-3p treated mice showing (A) elevated levels of AUF1 (p<0.023) and decrease levels of FOXO-1, IL-1-β, p21 and p16 compared to vehicle control. The quantification analysis of western blot (B) FOXO-1 (p<0.009), (C) AUF1 (p<0.023), (D) IL-1β (p<0.096), (E) p21 (p<0.001) and (F) p16 (p<0.001) using muscle lysates from anti-miR-141-3p and vehicle control treated animals. Data are expressed as mean ± SEM. Data were analyzed by student’s t test for two groups or ANOVA for more than two groups (n=8-9/group *p<0.05, **p<0.01, #p<0.001).

Bioinformatics analysis revealed that miRNA-141-3p has a binding site for RNA-binding protein AUF1. To confirm this, C2C12 cells were treated with miR-141-3p mimic, and a western blot was performed on AUF1. C2C12 cells treated with miR-141-3p mimic significantly (p<0.01) decrease the expression of AUF1. (Fig. 5A, Fig. 5B). Surprisingly, we found that the expression of Forkhead transcription factors (FOXO-1), a known muscle wasting transcription factor FOXO-1, increased (p<0.001) in miR-141-3p treated group (Fig. 5G, Fig. 8B). To investigate the connection between miR-141-3p, AUF-1, and FOXO-1 gene regulation, we performed several *in vitro* experiment’s and validated our findings in muscle lysate of anti-miR-141-3p treated mice. We transfected C2C12 cells with miR-141-3p-mimic and AUF1 siRNA and performed a western blot for FOXO-1 expression. We found that both the miR-141-3p-mimic (p<0.001) and silencing AUF1 (p<0.001) enhanced significantly the FOXO-1 expression (Fig. 5H, Fig. 5K). We also examined AUF1 and FOXO-1 expression in muscle lysate on Anti-miR-141-3p treated mice. We found that AUF1 expression increased significantly (p<0.023) (Fig. 6A, Fig. 6C), and FOXO-1 expression was decreased (p<0.009) (Fig. 6A, Fig. 6B), consistent with our *in vitro* findings. It is well known that AUF1 regulate the expression of several SASP and cytokines [32,33]. To investigate this, we checked the expression of pro-inflammatory cytokines and found that IL-1β expression was augmented (p<0.001) in AUF1 siRNA treated cells (C2C12) as compared to control (Fig. 5C, Fig. 5D). Similarly, the expression of senescence markers such as p16 (p<0.011) and p21 (p<0.001) was augmented in AUF1 siRNA-treated cells as compared to the control (Fig. 5C, Fig. 5E, Fig. 5F). Further proving that AUF1 is a critical player in maintaining cytokine stability and suggesting that it could be beneficial in reducing inflammation with advancing age.

## DISCUSSION

We previously demonstrated elevated levels of miR-141-3p with age in human and murine bone marrow environments [34]. We have also reported that miR-141-3p inhibits the osteogenic differentiation of BMSCs by targeting SDF1 and SVCT2 [20]. Several other groups have shown elevated levels of miR-141-3p in age-related diseases, such as neurodegenerative disorders [33], cardiovascular diseases [22, 36], and diabetic/metabolic [37]. Zheng et al. demonstrated that augmented expression of miR-141-3p resulted in elevated oxidative stress, mitochondrial dysfunction, and cells undergoing apoptosis [38]. The study performed by Gayen et al. demonstrated higher levels of miR-141-3p in the exosomes of astrocytes stimulated by inflammation [39]. A recent study found that inhibition of miR-141-3p improved mortality, neurological deficits, and decreased infarct volumes in aged animals [36]. The current study was designed to answer the critical question whether by inhibiting miR-141-3p during aging can we improve overall health, specifically systemic and musculoskeletal health. For this study, we treated mice at 15 months of age subcutaneously twice a week for three months. Our group [40] and others reported that mice systemic health (elevated oxidative and inflammatory stress) and musculoskeletal health started to decline after 15 months [41, 42].

Overall health depends on oxidative and inflammatory stress levels in tissue and organs. With advanced age, pro-inflammatory cytokines tend to be higher [2]. In our study, we found that serum pro-inflammatory cytokine levels (TNF-α, IL-1β, IFN-γ) declined in the Anti-miR-141-3p treatment. Moreover, we found decreased M1 and increased M2 macrophage in splenocytes in the treated group. This suggested that Anti-miR-141-3p treatment helps in maintaining better immune health. Furthermore, we also investigated the M1/M2 macrophage in the muscle section using immunofluorescent staining. Our results demonstrated that anti-miR-141-3p enhanced the M2 macrophage population (increase CD206). The study performed by Cui et al. showed that M2 was the major type of macrophage in healthy human skeletal muscle compared to M1 [43]. Another study performed by Verma et al. showed that inhibition 141-3p miRNA restored the decreased expression of M2 macrophages and augmented IL-6 activity, thus enhancing anti-inflammatory potential [44]. M2 macrophages have been shown to play a role in tissue repair by suppressing inflammation and promoting collagen synthesis [45]. Therefore, based on our findings, we suggest that augmentation in the M2 macrophage population instigated by anti-miR-141-3p promotes overall musculoskeletal health and healthy aging.

**Figure 7.**
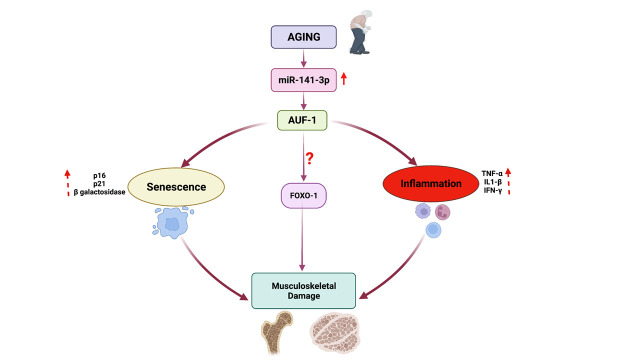
Schematic diagram showing involvement of microRNA-141-3p signaling in age-related musculoskeletal health.

Maintaining musculoskeletal health is an important contributing factor to healthy aging and longevity [46, 47]. We, therefore, analyzed the effects of Anti-miR-141-3p on muscle and bone microstructure. We observed muscle fiber size was increased in the mice treated with Anti-miR-141-3p compared to the vehicle-treated group. Our study's findings are consistent with the study conducted by Lee et al. Their study reported a decline in muscle mass and mitochondrial function in the post-menopausal (ovariectomized) mice and elevated levels of miR-141-3p. Inhibition of miR-141-3p augmented mitochondrial respiration and enhanced myogenesis in C2C12 cells [23]. As expected, we also observed better bone microstructure (BMD, trabecular separation, trabecular number, bone surface) in Anti-miR-141-3p treated animals. Previously, we reported that inhibition of miR-141-3p helps osteogenic markers and differentiation of BMSCs. Several studies reported similar findings that overexpression of miR-141-3p has a negative effect on osteogenic differentiation [48, 49] and bone regeneration [50]. Ours is the first study to demonstrate the treatment with Anti-miR-141-3p decreases systemic inflammation and improves musculoskeletal health in aged mice.

To better understand the molecular mechanism behind the overall positive effect of Anti-miR-141-3p on aging, we performed *in-vitro* studies on C2C12. We hypothesized that miR-141-3p induces a determinate effect on aging cells through multiple signaling pathways, specifically by promoting a pro-inflammatory environment and inducing premature senescence and vice-versa. Our data demonstrated that treatment of C2C12 cells with miR-141-3p elevated the expression of pro-inflammatory cytokines (TNF-α, IL-1β) and senescence markers (p16, p21) and Anti-miR-141-3p reversed the effect. It is well known that p16 and p21 expression increases with age in senescence cells and [51-53] in several musculoskeletal tissues [54, 55]. The bioinformatics analysis of miR-141-3p and pro-inflammatory factors and senescence (p16, p21) genes did not show a direct interaction. So, we hypothesize that miR-141-3p might indirectly regulate the expression of the above-mentioned genes.

Previous studies demonstrated that several RNA-binding proteins regulate the stability and translation of some pro-inflammatory cytokine transcripts [56, 57]. Considering this, we performed bioinformatics analysis to predict if miRNA-141-3p would bind to the RNA-binding proteins 3′UTR. Among the candidates, AU-binding factor 1 (AUF1) was selected for further study. Previously, Al-Khalaf and the group demonstrated a direct relation between miR-141-3p and AUF1 (AUF1 3’UTR has a binding site for miR-141-3p) using luciferase activity [58]. We hypothesize that miR-141-3p might regulate pro-inflammatory factors and senescence markers (p16, p21) through AUF1. This is indeed the case. Transfection of miR-141-3p decreased the expression of AUF1, which validated the previous findings [59]. Most importantly, the expression of pro-inflammatory IL-1β was up-regulated in AUF1 siRNA-treated cells, emphasizing that AUF1 is a critical player in regulating cytokine. Moreover, the expression of senescence markers (p16 and p21) was augmented in AUF1 siRNA-treated C2C12 cells. We validated some of the genes in the muscle lysate of Anti-miR-141-3p treated animals. We found elevated levels of AUF1 and decreased p16, p21, TNFα, and IL-1β. Several studies demonstrated AUF1 regulated expression of above mention genes in the animal model [58, 60-63] and *in vitro* studies [64]. These genes (p16, p21, TNFα, and IL-1beta) have one or more AU-rich elements (AREs) in their 3′-untranslated regions. The binding of AUF1 to AU-rich elements to these genes 3’UTR region is responsible for the degradation of mRNA transcripts [65]. In our study, a decrease in inflammation and senescence markers after Anti-miR-141-3p treatment might be due to elevated levels of AUF1. Anti-miR-141-3p prevents degradation of AUF1 by inhibiting the miRNA-141, allowing AUF1 to bind inflammatory and senescence markers and decay mRNA or prevent translation (Fig. 7).

In this study, we also analyzed the transcription factor FOXO-1, known for its regulatory role in muscle biology. Several studies demonstrated that FOXO-1 elevated with age in muscle [66, 67]. We observed a decreased FOXO-1 expression with anti-miR-141-3p treatment in the muscle and elevated levels (of FOXO-1) in C2C12 cells when treated with miR-141-3p mimic. Furthermore, we observed a decreased FOXO-1 expression with Anti-miR-141-3p and AUF1 siRNA, suggesting some crosstalk. The molecular mechanism of how FOXO-1 expression is regulated by the miR-141-3p-AUF1 axis is an area that needs further assessment.

To our knowledge, this is the first study to show the therapeutic application of Anti-miR-141-3p to delay age-related pathological changes and promote healthy aging. We showed that inhibiting miR-141-3p decreased the burden of inflammatory cytokines and improved bone and muscle health. We demonstrated the involvement of the miR-141-3p-AUF1 axis In-vivo and In-vitro-dependent inflammatory and senescence processes. Overall, our study showed that inhibiting miR-141-3p could be beneficial in delaying the aging process and a promising potential strategy to promote healthy aging.

## Supplementary Materials

The Supplementary data can be found online at: www.aginganddisease.org/EN/10.14336/AD.2023.0310-1.

## Data Availability

The datasets generated during and/or analysed during the current study are available from the corresponding author on reasonable request.
