# Supplementary material for: Inhibiting MicroRNA-141-3p Improves Musculoskeletal Health in Aged Mice
Source: Aging Dis. 2023 Dec 1;14(6):2303–16. doi: 10.14336/AD.2023.0310-1 (PMC10676793; doi:10.14336/AD.2023.0310-1)
Supplement: Supplementary file 1 — The Supplementary data can be found online at: www.aginganddisease.org/EN/10.14336/AD.2023.0310-1. [file AD-14-6-2303-s.pdf]

SUPPLEMENTARY DATA

Inhibiting MicroRNA-141-3p Improves Musculoskeletal Health in Aged Mice

Sagar Vyavahare[1#], Sandeep Kumar[1#], Kathryn Smith[2], Bharati Mendhe[1], Roger Zhong[3], Marion A. Cooley[4], Babak Baban[4], Carlos M. Isales[5,6,7], Mark Hamrick[1,6], William D Hill[8], Sadanand Fulzele[1,5,6,7*]

© 2023. Vyavahare S et al. Published online at http://www.aginganddisease.org/EN/10.14336/AD.2023.0310-1

SUPPLEMENTARY DATA

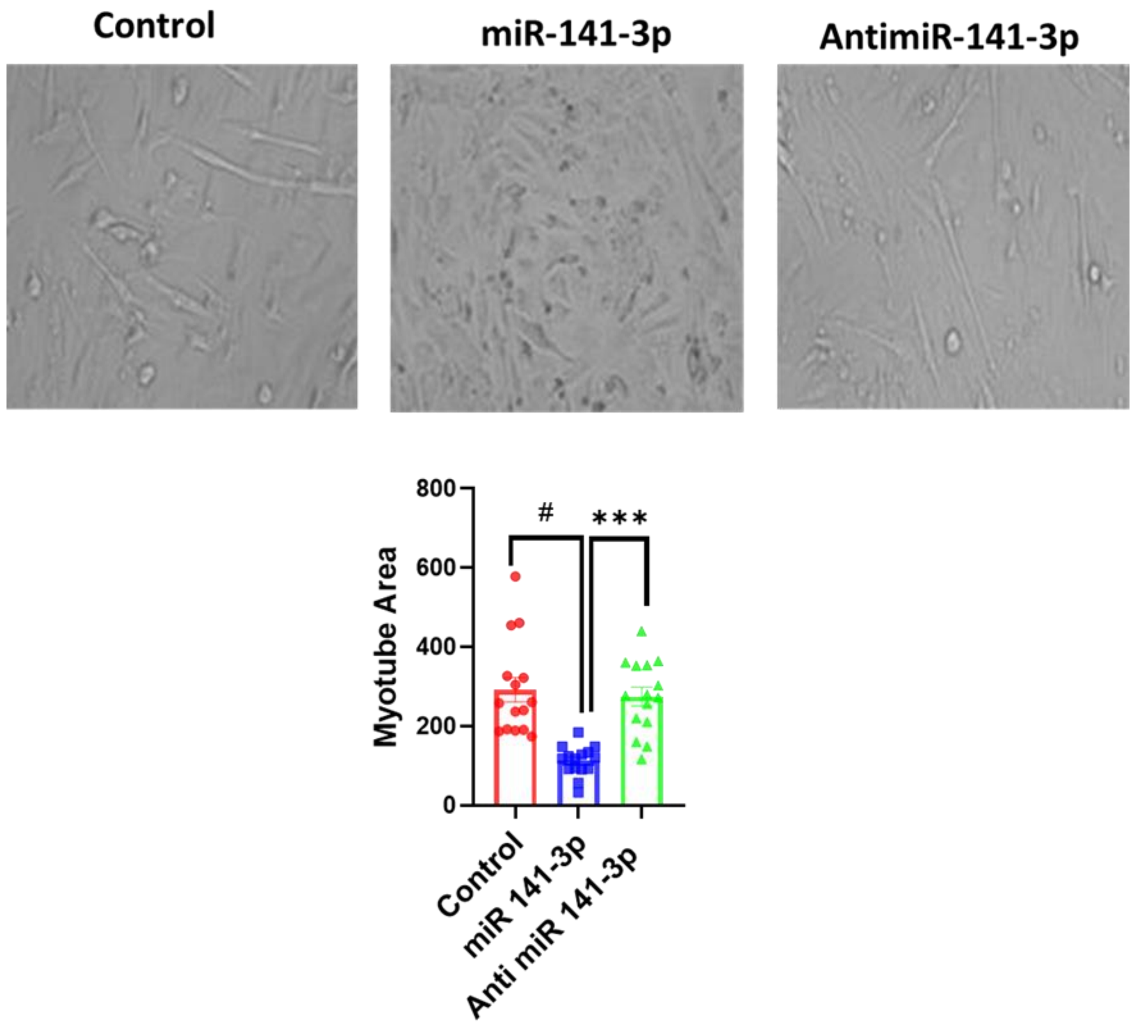

Supplementary Figure 1. MiR-141-3p regulates myotube formation. Myotube differentiation assay was performed to assess the role of mir-141-3p mimic and anti-miR-141-3p on C2C12 cells. Our results demonstrated that 141-3p mimic inhibited and anti-miR-141-3p showed improved myogenic differentiation/myotube formation.

© 2023. Vyavahare S et al. Published online at http://www.aginganddisease.org/EN/10.14336/AD.2023.0310-1
